# Supplementary material for: Cellular Internalization of Beta-Carotene Loaded Polyelectrolyte Multilayer Capsules by Raman Mapping
Source: Molecules. 2020 Mar 25;25(7):1477. doi: 10.3390/molecules25071477 (PMC7181117; doi:10.3390/molecules25071477)
Supplement: Supplementary file 1 [file molecules-25-01477-s001.pdf]

Supplementary information

Cellular internalization of beta-carotene loaded polyelectrolyte multilayer capsules by Raman mapping

Loredana F. Leopold[1], Oana Marișca[2*], Ioana Oprea[1], Dumitrița Rugină[2], Maria Suciu[3], Mădălina Nistor[1], Maria Tofană[1], Nicolae Leopold[4], Cristina Coman[1*]

Figures S1, S2, S3, S4 below show the first ten score and loading plots for all Raman mapping figures shown in the manuscript (Figure 2, 3, 5 and 6). Each figure contains also a graphical representation of the explained variance of the first ten PCs. As can be observed from the graphical representations, in each case the cumulative explained variance was over 90%.

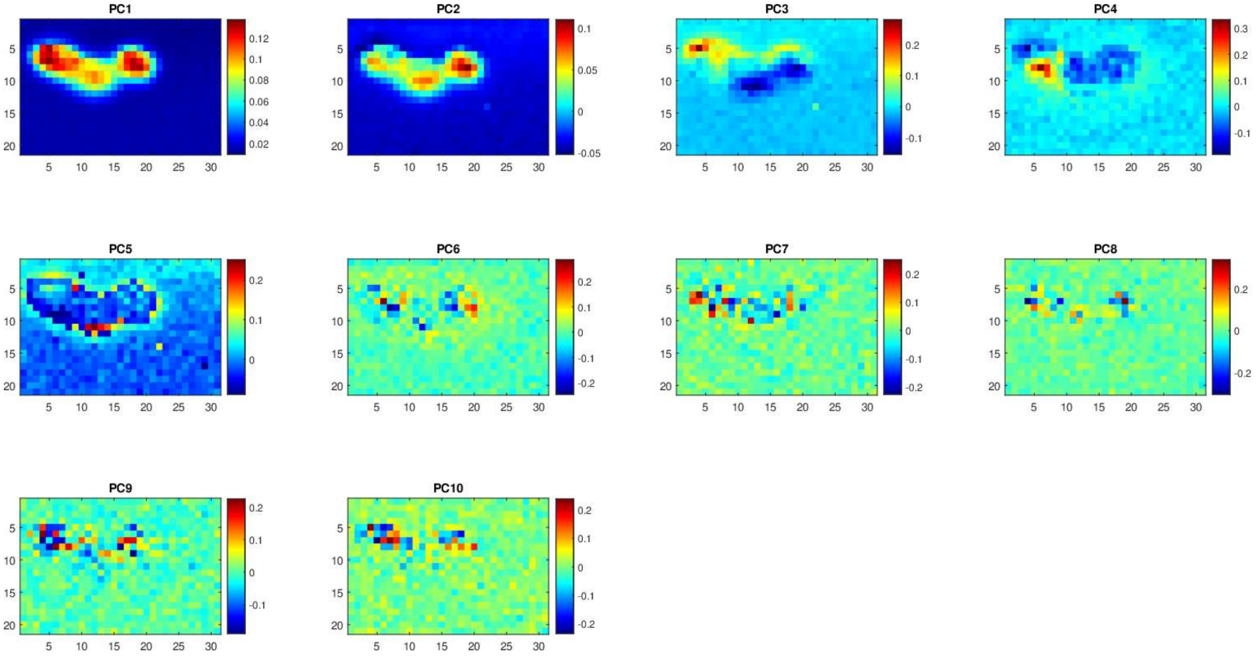

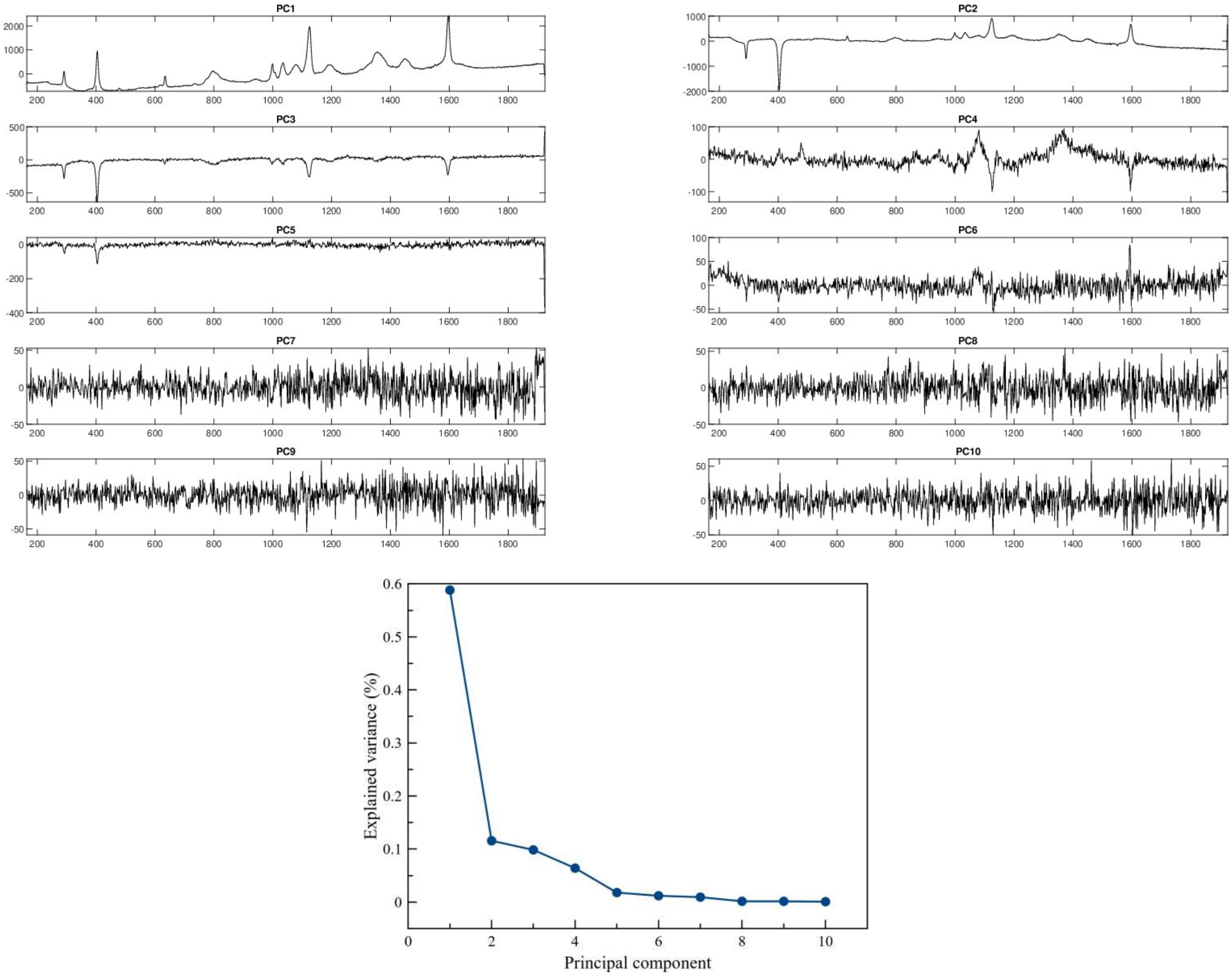

Figure S1. From top to bottom, score plot and loading plot of the first ten PCs of the PCA of the Raman map presented in Figure 2, and graphical representation of the explained variance of the first ten PCs.

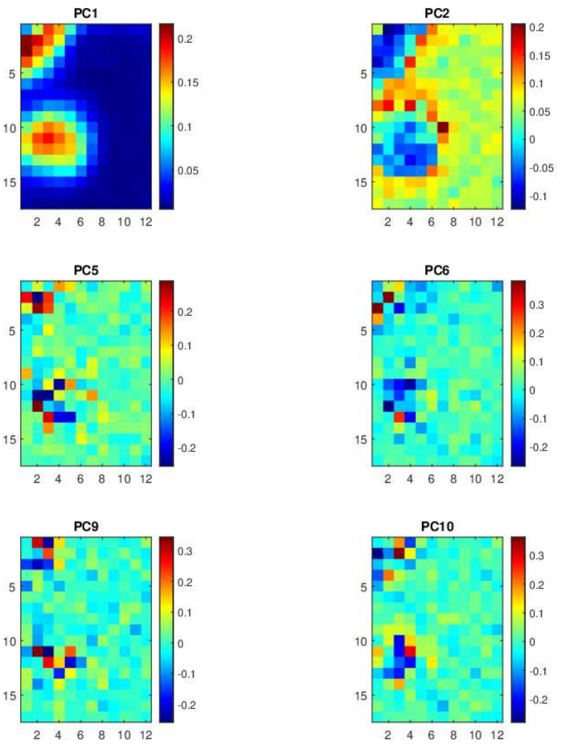
PC1
PC2
PC3
PC4
PC5
PC6
PC7
PC8
PC9
PC10

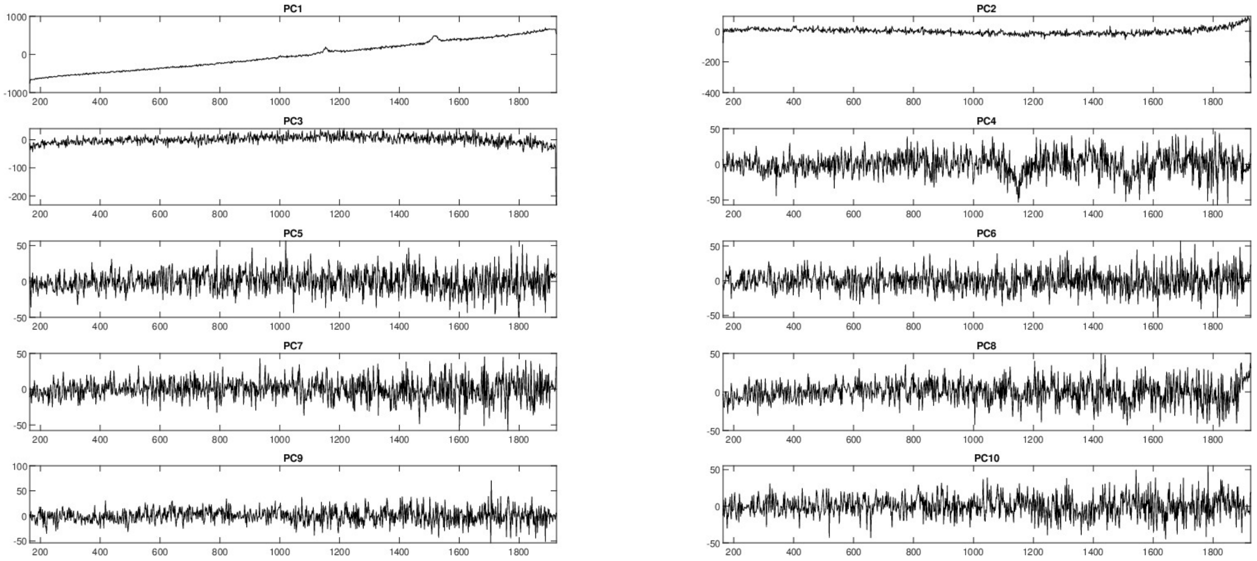
PC1
PC2
PC3
PC4
PC5
PC6
PC7
PC8
PC9
PC10

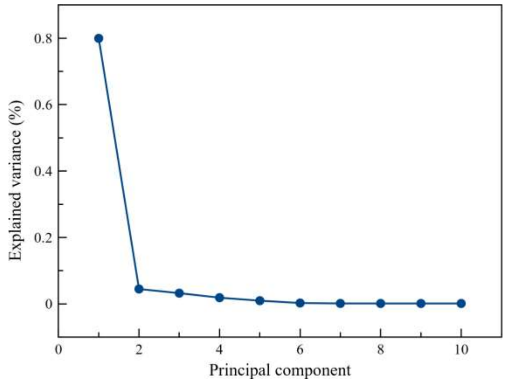

Figure S2. From top to bottom, score plot and loading plot of the first ten PCs of the PCA of the Raman map presented in Figure 3, and graphical representation of the explained variance of the first ten PCs.

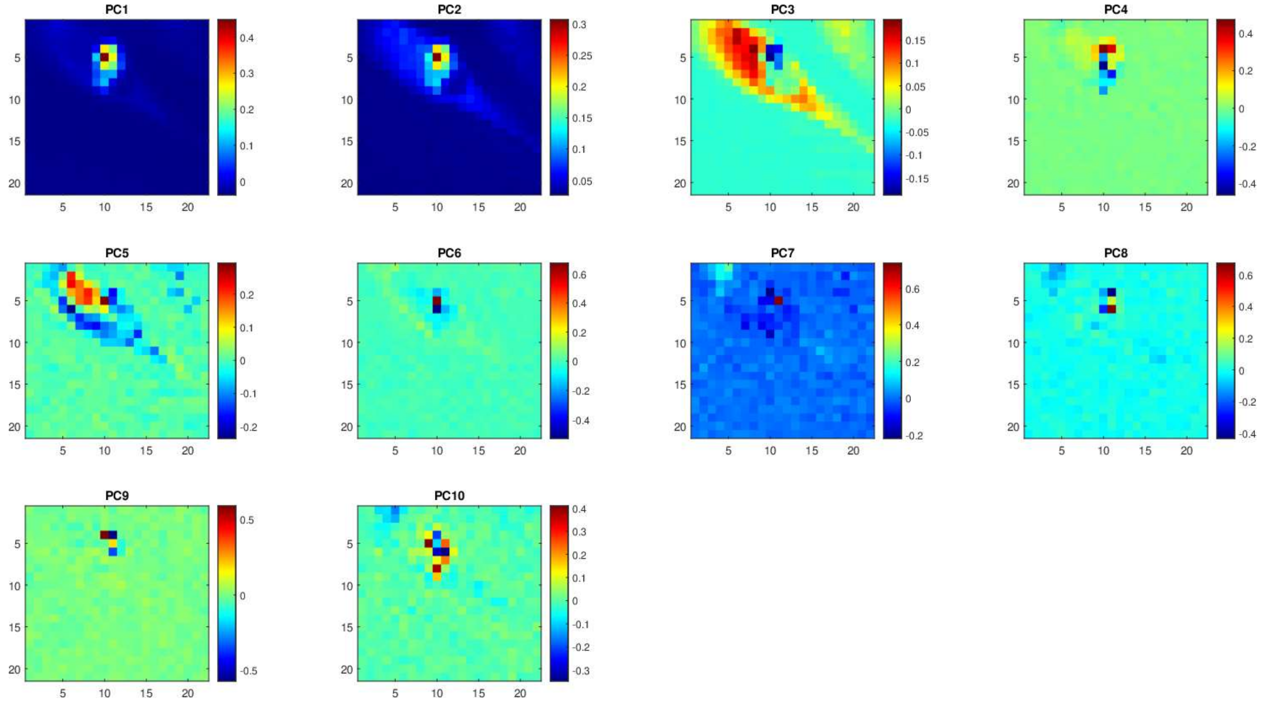

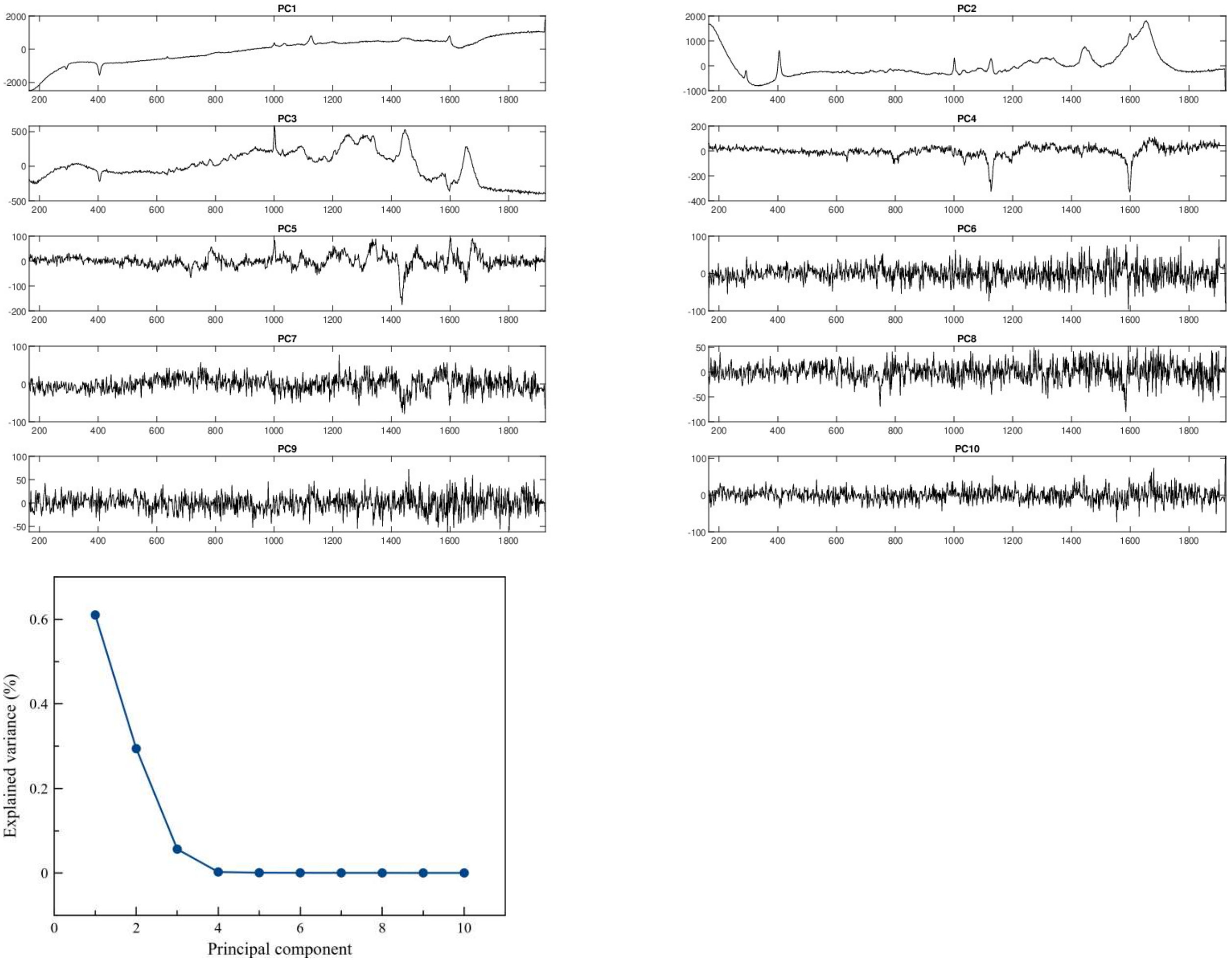

Figure S3. From top to bottom, score plot and loading plot of the first ten PCs of the PCA of the Raman map presented in Figure 5, and graphical representation of the explained variance of the first ten PCs.

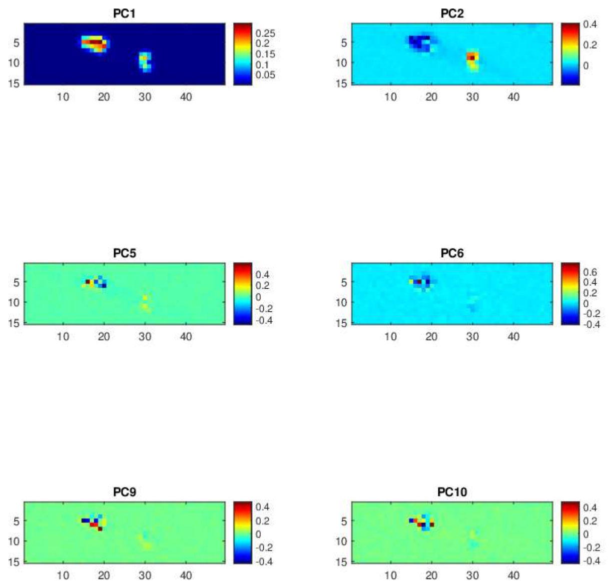
PC1
PC2
PC3
PC4
PC5
PC6
PC7
PC8
PC9
PC10

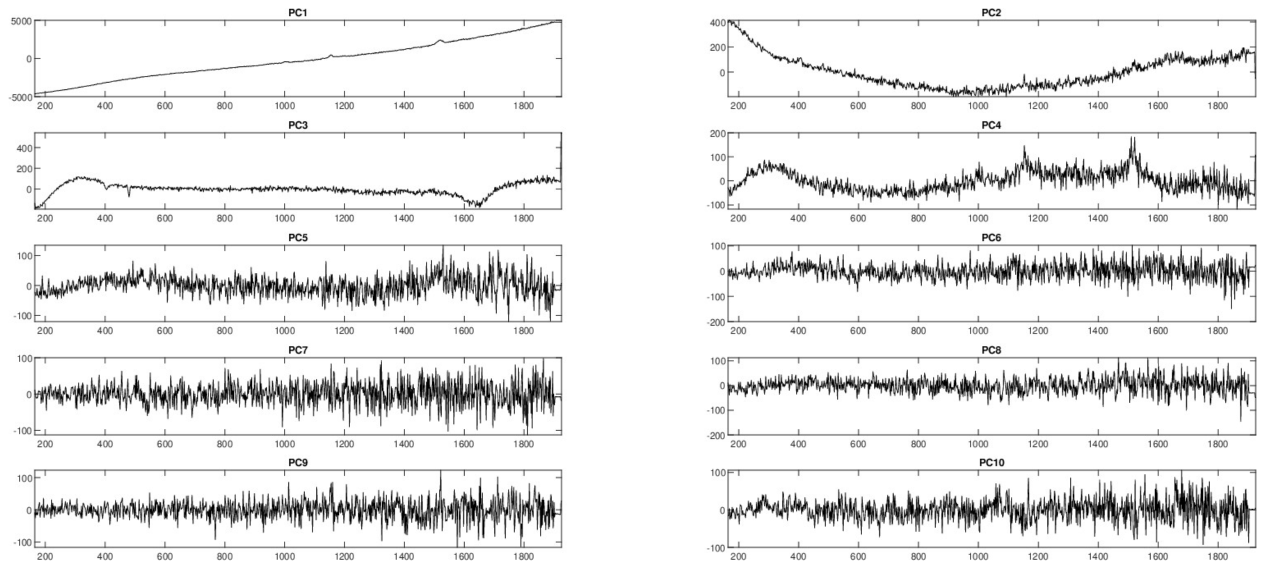
PC1
PC2
PC3
PC4
PC5
PC6
PC7
PC8
PC9
PC10

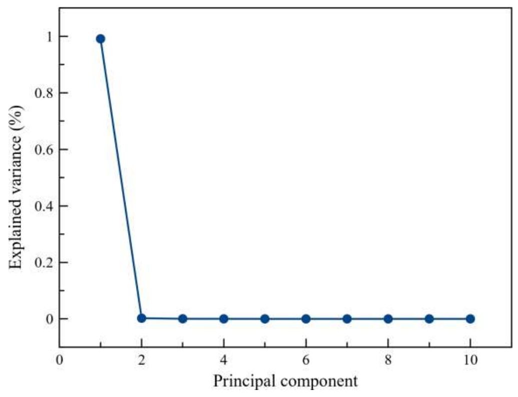
Explained variance (%)
Principal component

Figure S4. From top to bottom, score plot and loading plot of the first ten PCs of the PCA of the Raman map presented in Figure 6, and graphical representation of the explained variance of the first ten PCs.

To provide additional proof that our mapping results provide consistent information, below (Figures S5-S10) are shown additional mappings performed on independent assays than the ones shown in the manuscript (different than the ones shown in Figures 2,3,5,6). Each of the illustrated results leads to the same main conclusions presented in the manuscript.

Figures S5 and S6 show corresponding Raman score plots (maps) and loadings for the non-loaded capsules (Figure S5) and beta-carotene loaded capsules (Figure S6). Consistent with Figure 2 , the PC1 loading plots in Figure S5 contain the characteristic Raman bands of PSS/PAH polymers, at 1126 cm^{-1} and 1597 cm^{-1}. Regarding Figure S6, the loading plot PC1 shows the characteristic marker peaks of beta carotene marker at 1157 and 1520 cm^{-1}

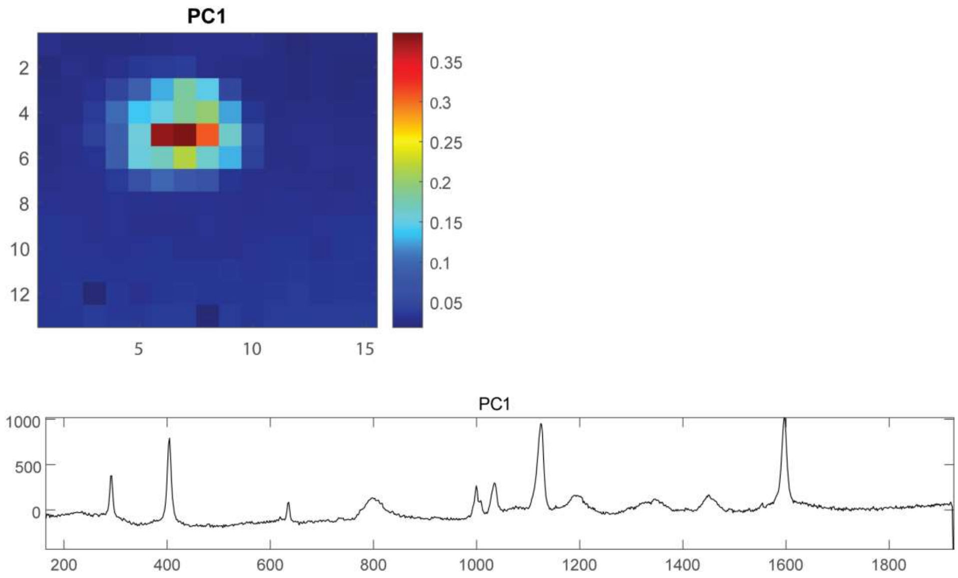

Figure S5. Top: Raman map of non-loaded microcapsules. Bottom: The corresponding loading plot of the relevant principal component PC1.

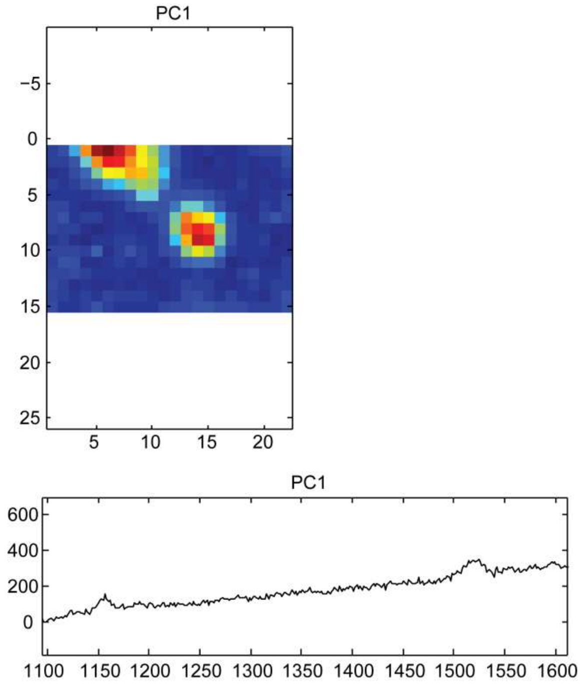

Figure S6. Top: Raman map of beta-carotene loaded microcapsules. Bottom: The corresponding loading plot of the relevant principal component PC1.

Figures S7 and S8 illustrate the most significant PCA components resulted by the Raman mapping analysis of the D407 cells exposed to non-loaded PEM capsules. Both the cells and the capsules can be identified in the corresponding Raman score plots (maps).

The PC2 and PC3 loading plots in Figure S7 contain mixed contributions from the polymers (1126 and 1598 cm^{-1}) and characteristic cell Raman bands (most pronounced at 1001, 1446, 1654 cm^{-1}), the cellular shape being best visible in the PC3 score plot. The capsule characteristic polymer peaks are best evidenced by the PC4 loading plot, while the PC5 plot shows primarily cellular characteristic peaks.

Regarding Figure S8, the PC1, PC2 and PC3 loading plots show mixed contributions from cellular components and polymers, while the PC4 loading plot and corresponding score plot show mostly contribution from the polymer vibrations.

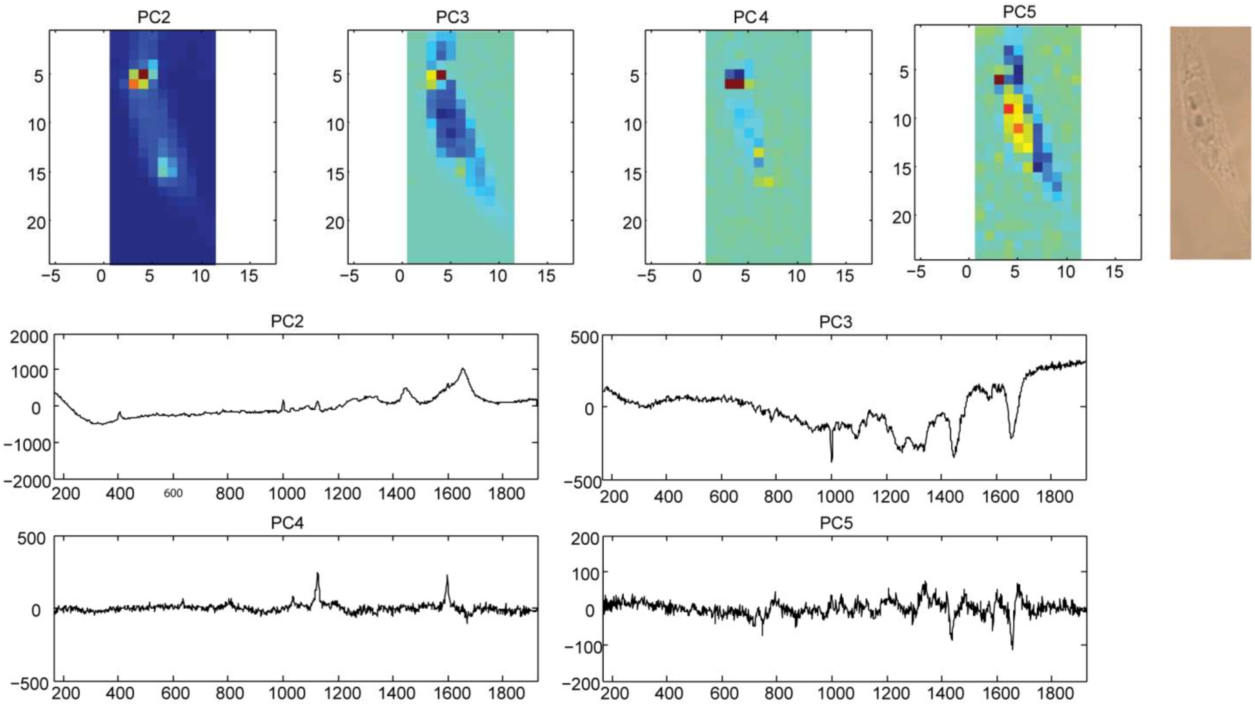

Figure S7. Top: Raman maps of a D407 cell transfected with non-loaded PEM capsules followed by the optical microscopy image of the cell. Bottom: The corresponding loading plots of relevant principal component PC2, PC3, PC4, and PC5.

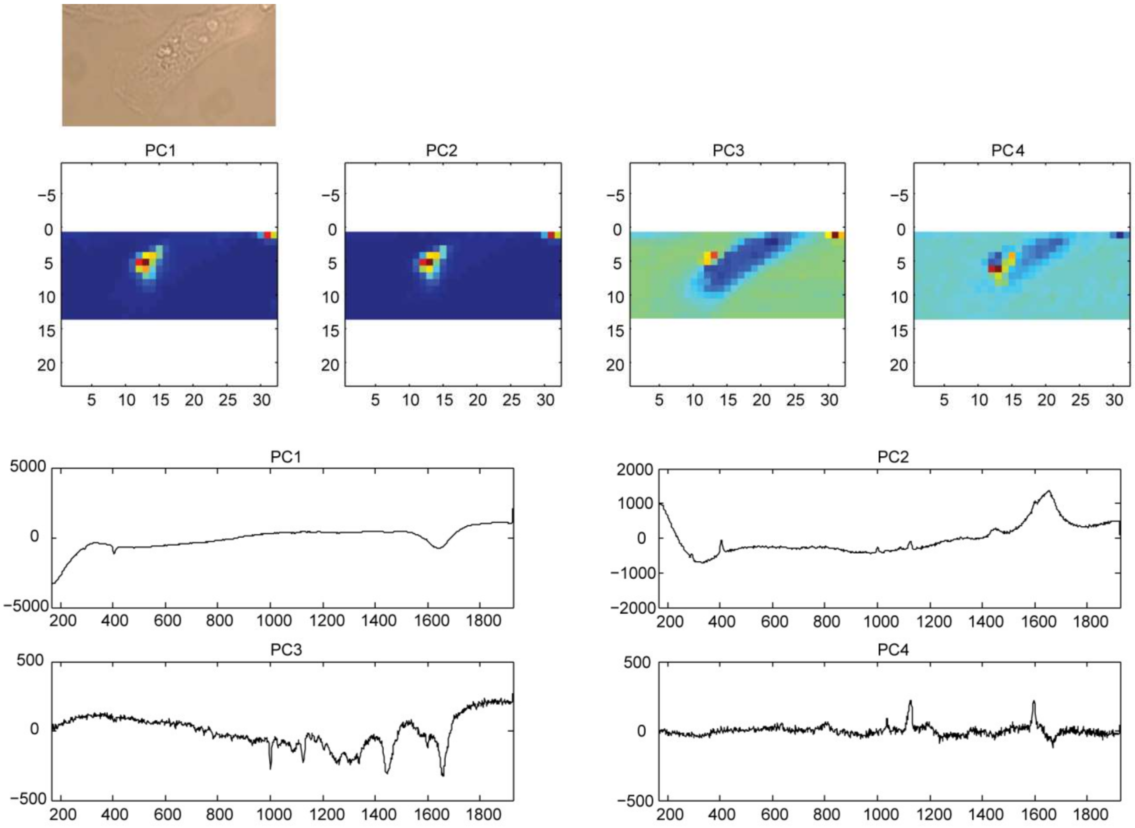

Figure S8. Top: Optical microscopy image of one D407 cell exposed to non-loaded PEM capsules. Middle: Corresponding Raman maps of the PC1, PC2, PC3 and PC4 components. Bottom: The corresponding loading plots of relevant principal component PC1, PC2, PC3, and PC4.

Figures S9 and S10 show characteristic Raman maps and corresponding score plots for D407 cells exposed to beta-carotene loaded capsules.

In Figure S9, the loading plots and corresponding score plots (PC1 and PC3) contain characteristic Raman fingerprint of the beta-carotene (1157 and 1517 cm^{-1}).

Regarding the Figure S10, mixed contributions from beta-carotene capsules and cells can be observed, the cellular shape being mostly visible in PC3.

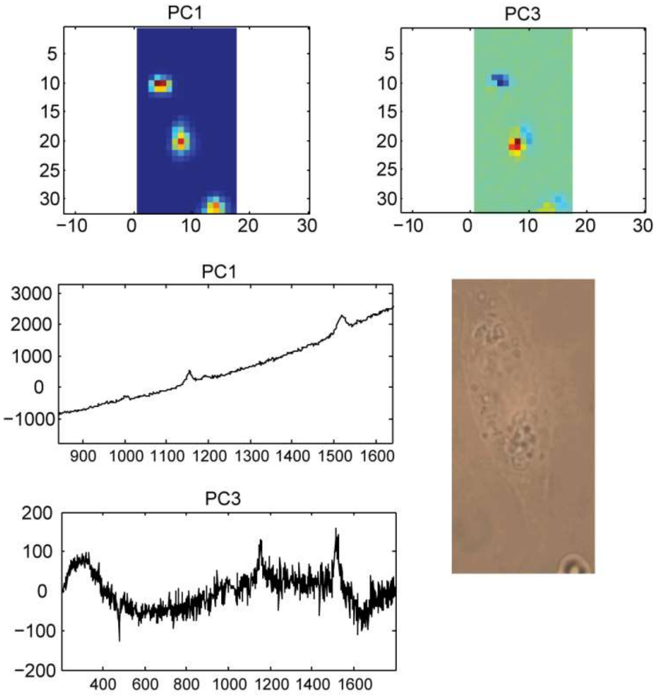

Figure S9. Top: Raman score plots (maps) and corresponding loading plots of relevant principal components PC1 and PC3 (bottom) of a D407 cell transfected with beta-carotene loaded PEM capsules. Bottom right: Optical microscopy image of the analyzed cell.

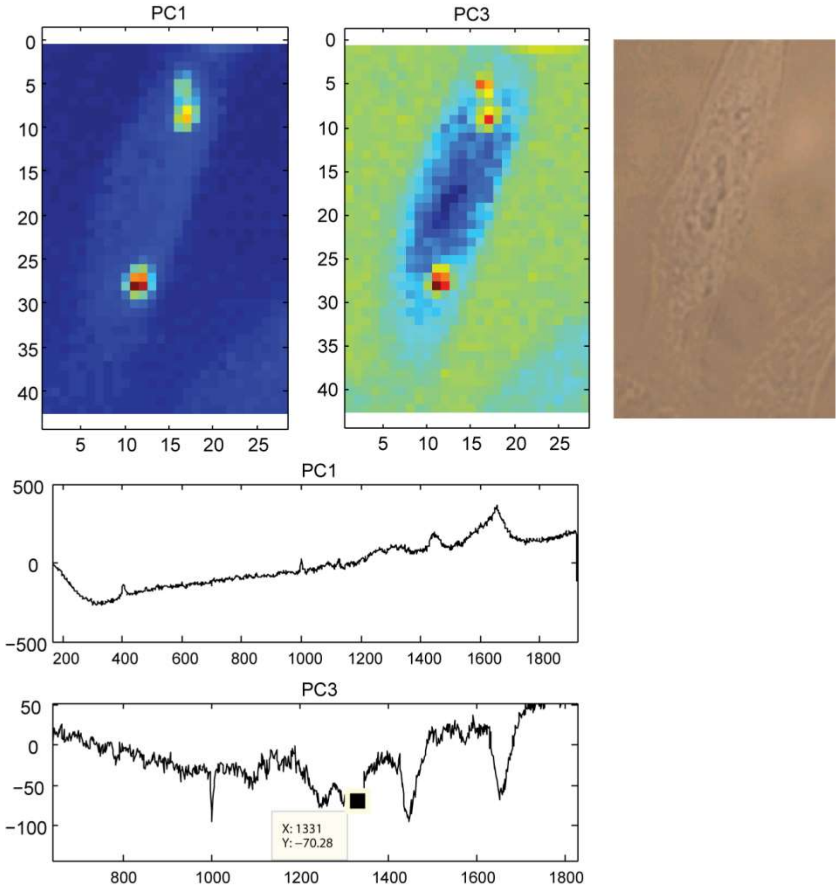

Figure S10. Top: Raman score plots (maps) of a D407 cell exposed to beta-carotene loaded PEM capsules, followed by the optical microscopy image of the cell on the right. Bottom: The corresponding loading plots of relevant principal components PC1 and PC3.
